# ENKIE: a package for predicting enzyme kinetic parameter values and their uncertainties

**DOI:** 10.1093/bioinformatics/btae652

**Published:** 2024-11-04

**Authors:** Mattia G Gollub, Thierry Backes, Hans-Michael Kaltenbach, Jörg Stelling

**Affiliations:** Department of Biosystems Science and Engineering and SIB Swiss Institute of Bioinformatics, ETH Zurich, 4056 Basel, Switzerland; Department of Biosystems Science and Engineering and SIB Swiss Institute of Bioinformatics, ETH Zurich, 4056 Basel, Switzerland; Department of Biosystems Science and Engineering and SIB Swiss Institute of Bioinformatics, ETH Zurich, 4056 Basel, Switzerland; Department of Biosystems Science and Engineering and SIB Swiss Institute of Bioinformatics, ETH Zurich, 4056 Basel, Switzerland

## Abstract

**Motivation:**

Relating metabolite and enzyme abundances to metabolic fluxes requires reaction kinetics, core elements of dynamic and enzyme cost models. However, kinetic parameters have been measured only for a fraction of all known enzymes, and the reliability of the available values is unknown.

**Results:**

The ENzyme KInetics Estimator (ENKIE) uses Bayesian Multilevel Models to predict value and uncertainty of $K_{M}$ and $k_{\mathrm{cat}}$ parameters. Our models use five categorical predictors and achieve prediction performances comparable to deep learning approaches that use sequence and structure information. They provide calibrated uncertainty predictions and interpretable insights into the main sources of uncertainty. We expect our tool to simplify the construction of priors for Bayesian kinetic models of metabolism.

**Availability and implementation:**

Code and Python package are available at https://gitlab.com/csb.ethz/enkie and https://pypi.org/project/enkie/.

## 1 Introduction

To construct dynamic mathematical models for metabolic networks (Kim *et al.* 2018) or to relate reaction fluxes to metabolite and enzyme abundances in protein cost models (Noor *et al.* 2016), a main challenge is to find kinetic parameter values for $k_{\mathrm{cat}}$ (rate constants) and $K_{M}$ (Michaelis constants). Kinetics databases such as BRENDA (Chang *et al.* 2021) (parameter values for ∼90 000 enzymes from >150 000 publications) and SABIO-RK (Wittig *et al.* 2018) (∼7500 publications) cover only a minority of known enzymes (e.g. in few species).

To predict parameter values from existing databases, a recent combination of machine- and deep-learning models achieved a coefficient of determination (*R*^2^) of 0.53 for affinities (Kroll *et al.* 2021) and of 0.44 for catalytic rates (Kroll *et al.* 2023) on independent test sets. Unfortunately, R^2^ (or RMSE) values are dataset-specific, and accuracy is not guaranteed for other data, e.g. with different overlap with the training data. The uncertainty of measured values is also poorly characterized: most parameters have few measurements. For reliable uncertainty quantification in kinetic models of various pathways and organisms, uncertainties of measured and predicted values should be computed per parameter.

Here, we develop Bayesian Multilevel Models (BMMs) that capture a simple hierarchy of coarse-grained enzyme properties to predict $K_{M}$ and $k_{\mathrm{cat}}$ values as well as their uncertainties without considering structural and physical properties of enzymes, reactions, and metabolites explicitly. The models can be easily used through ENKIE, a python package for the estimation of thermodynamically consistent enzyme kinetic parameter values and their uncertainties.

## 2 Materials and methods

*Statistical models*. We use hierarchical BMMs to express the relationship between the properties of reactions/enzymes/metabolites and the value of a kinetic parameter $K_{M}$ or $k_{\mathrm{cat}}$ (Supplementary Methods). The BMMs linearly combine *population-level* effects (with a single value for the entire population) and *group-level* effects (with different values across groups and a common normal distribution). They capture the reduction in variability as the classification of a parameter value becomes more precise. For $K_{M}$ and $k_{\mathrm{cat}}$ values, we assume normally distributed residuals with mean $\mu_{*}$ and standard deviation $\sigma_{*}$ (∗∈{M,cat}). We describe $\mu_{*}$ by a population average and a series of nested group-level effects. For $K_{M}$, the substrate is the first grouping level because parameter values for a substrate are conserved across reactions (Park *et al.* 2016). We then nest the Enzyme Commission (EC)-reaction pair, the protein family, and the protein identifier denoting a specific enzyme of a specific organism. For $k_{\mathrm{cat}}$, we replace the substrate with a nested hierarchy of the first three components of the EC number and include the reaction direction to distinguish between forward and backward rates. To model $\sigma_{*}$, we use two different population averages for entries with/without an annotated protein identifier because entries with unknown identifier are presumably harder to fit. We use a group-level effect to incorporate that the size of the residuals can differ across reactions because residuals capture many unmodeled factors, such as experimental errors, different experimental conditions, and annotation errors.

*Fitting and evaluation*. The BMMs capture model uncertainty (confidence in the estimated model parameters given the data) and the residuals $\sigma_{*}$ (discrepancy between measurements of the same parameter, possibly because of unmodeled effects). Because these can be strongly correlated, we fitted the models in a Bayesian framework. Specifically, we implemented the models in R and fitted them using the Markov Chain Monte Carlo (MCMC)-based brms package (Bürkner 2017) to 94’968 entries extracted from BRENDA and SABIO-RK (Supplementary Methods). The simulated chains had no divergent transitions and a potential scale reduction factor $\hat{R}\leq 1.02$, suggesting good fit quality (Gelman *et al.* 2013) (Supplementary Table S7).

*Parameter balancing*. Constraints by e.g. thermodynamics induce dependencies between kinetic parameters. Optionally, ENKIE estimates free energies with eQuilibrator (Beber *et al.* 2022). Parameter balancing (Lubitz *et al.* 2010) combines them with samples from the joint distribution of predicted parameters to obtain a multivariate normal distribution of thermodynamically consistent estimates and uncertainties of free energies and kinetic parameters. For model analysis, we omitted this step to simplify benchmarking. (see Supplementary Methods).

**Figure 1.**
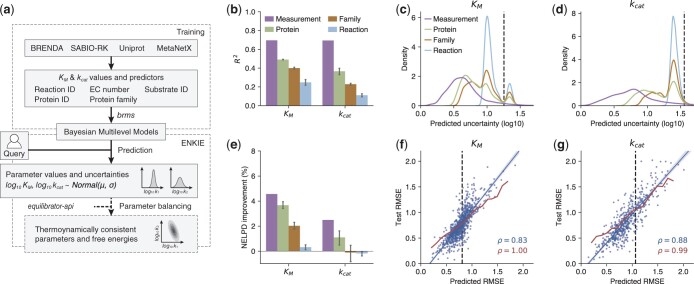
ENKIE design and performance. (a) Overview. We gather kinetic parameter values from BRENDA and SABIO-RK, use Uniprot and MetaNetX to consistently annotate them, and train models for value and uncertainty of $K_{M}$ and $k_{\mathrm{cat}}$ parameters. The ENKIE package uses the models to predict value and uncertainty for query parameters, optionally with eQuilibrator (Beber *et al.* 2022) and parameter balancing (Lubitz *et al.* 2010) to construct thermodynamically consistent parameters. (b) $R^{2}$ for ENKIE predictions of parameter values for measurements, proteins, protein families, and reactions outside of the training set. (c, d) Distribution of predicted parameter uncertainties on different dataset splits. Dashed lines: SD of parameter values over the entire dataset. (e) Improvement in the Negative Expected Log Predictive Density (NELPD) score for uncertainties predicted by ENKIE over the test RMSEs. (f, g) Predicted versus test $K_{M}$ (f) or $k_{\mathrm{cat}}$ (g) RMSEs binning the test data by metabolite (f, blue), reaction (g, blue) or predicted uncertainty (f, g, red). Correlations: Pearson’s ρ; dashed lines: RMSEs of the entire test dataset.

*Implementation*. ENKIE (Fig. 1a) uses generally available inputs: reaction stoichiometries, metabolite/reaction identifiers in any namespace supported by MetaNetX (Moretti *et al.* 2021), ECs, protein identifiers and, optionally, physiological properties of the reaction compartments (pH, pMg, temperature and ionic strength). It uses MetaNetX to standardize identifiers and Uniprot (The UniProt Consortium 2021) to obtain protein family annotations. Inputs are passed to the brmspredict method via the rpy2 package.

## 3 Results

We first analyzed the features’ contributions to the predicted $K_{M}$ and $k_{\mathrm{cat}}$ values (Supplementary Table S1). All population- and group-level effects were estimated with small uncertainty. The strongest determinants (i.e. the group-level effects with the largest average size) for $K_{M}$ and $k_{\mathrm{cat}}$ were the substrate and reaction identifier respectively. Importantly, the standard deviations of the only organism specific group-level effects (the Uniprot identifier, Supplementary Table S1) were significantly smaller than the standard deviations over the entire dataset (Supplementary Table S5). Hence, measurements from characterized organisms are informative for predicting values in new organisms.

In terms of training data quality, the average values of the estimated residuals $\sigma_{M}$ and $\sigma_{\mathrm{cat}}$ were slightly larger with missing protein identifier annotations (Supplementary Table S1), justifying the split into two groups. For entries with a protein identifier (which we expect to be always available in practical applications), estimated upper bounds of the 95% Confidence Intervals above one imply that, for certain parameters, multiple measurements with the same protein span several orders of magnitude.

Next, we characterized the predictive performance of our BMMs for completely unknown measurements, proteins, protein families, and reactions. Specifically, we performed 5-fold cross-validation with different types of folds (equally sized partitions of the input dataset; Supplementary Methods and Supplementary Fig. S2). The models remain predictive even when extrapolating to new reactions (Fig. 1b). Grouped folds are notoriously challenging and a drop in performance is expected. $K_{M}$ models generally achieve a better *R*^2^ than $k_{\mathrm{cat}}$ models. This could be because affinities are more conserved across organisms than rates, as shown by the lower variance explained by protein effects in $K_{M}$ models (13.2%) than in $k_{\mathrm{cat}}$ models (23.9%) (Supplementary Table S1). We compared the *R*^2^ for predicting $K_{M}$ and $k_{\mathrm{cat}}$ achieved by BMMs to state of the art Gradient Boosting (GB) approaches ([Bibr btae652-B7][Bibr btae652-B8]) using the training and test data of these GB publications. Our BMMs’ *R*^2^ scores ($K_{M}$: 0.46, $k_{\mathrm{cat}}$: 0.36) are only marginally lower than those of GB methods ($K_{M}:0.53$, $k_{\mathrm{cat}}$: 0.44). This is surprising because our models only use ECs, identifiers, and protein family annotations.

We characterized the BMMs’ predicted uncertainties first by their distributions (Fig. 1c and d). Uncertainties are low when the training set contains measurements for a parameter; they increase when training set and predicted parameter share less information. Without measurement, multiple peaks arise when only some test parameters had information about lower levels of the hierarchy (e.g. protein family); relying on higher-level information (e.g. reaction) increases uncertainties. Thus, ENKIE predicts uncertainties for varying overlaps with the training data.

To measure the quality of combined predictions of parameter values and uncertainties, we used the Negative Expected Log Predictive Density (NELPD) (Supplementary Methods), which quantifies the likelihood of the test data in the predicted distributions. Notably, the BMMs’ predicted uncertainties were generally more accurate than RMSEs computed on the test data, which represent the true error distribution in a set of predictions by an average (Fig. 1e). To make such NELPD improvements more tangible, we re-trained the models using random folds. This reflects real use cases where query parameters have different degrees of overlap with the training data (Supplementary Table S8). After binning the test data points by predicted uncertainties, the predicted and effective RMSEs match well for both $K_{M}$ and $k_{\mathrm{cat}}$ (Fig. 1f and g). Also, predicted uncertainties and actual errors for each metabolite (reaction) for $K_{M}$ ($k_{\mathrm{cat}}$) show strong correlations. By contrast, the constant test RMSE frequently over- or under-estimates prediction errors. Overall, ENKIE’s predicted uncertainties are well calibrated.

Lastly, we examined the group-level effects of the residuals to determine if there are systematic differences across reactions and organisms, hypothetically due to difficulties in quantifying specific compounds, the speed of reactions, differences across kingdoms, or annotation inaccuracies. We used the already fitted models and additionally fitted models for $K_{M}$ and $k_{\mathrm{cat}}$ where the group-level effects of the residuals were based on the organism (Supplementary Methods). Supplementary Figure S4 shows clear differences among reaction groups. For example, pyruvate kinase (MNXR103371) had a large positive effect for the residuals of $k_{\mathrm{cat}}$, caused by a single publication contributing 52 measurements for the same protein with strong variations in the concentrations of several ions and sugar phosphates. In contrast, the RuBisCO carboxylase and oxygenase reactions (MNXR191185 and MNXR191183) had negative effects for both $K_{M}$ and $k_{\mathrm{cat}}$, suggesting that the enzyme has been characterized with particular precision. For organism effects, we did not observe systematic differences across kingdoms (Supplementary Fig. S4).

## 4 Discussion

We build on linear regression to predict $K_{M}$ values (Borger *et al.* 2006), but introduce group-level effects. This enables prediction qualities comparable to current deep-learning methods that additionally use enzyme sequence and structure information. Importantly, ENKIE requires only the training data to provide reliable uncertainty estimates independent of the composition of the query. This makes it suitable for predicting parameter distributions for kinetic models of different pathways and organisms.

Compared to deep learning approaches, our models are easily interpretable. The effect sizes of the fitted models directly inform us about how much variance is explained by simple features such as substrate, reaction and protein family. We also showed how the effects on the residuals can be directly explained with the data. This is important because, in addition to ambiguously defined parameters, we frequently encountered challenges in the standardization and interpretation of database values. The residuals estimated by our Bayesian Multilevel Models (BMMs) highlight inconsistencies and can thus help improve the structure and accuracy of enzyme databases.

We addressed the challenge of uncertainty quantification through BMMs because their estimation methodology is well established. Uncertainties can in principle be computed in Bayesian deep learning as well, but this requires developments beyond the current approximate methods (Papamarkou *et al.* 2024). Compromising on prediction accuracy, ENKIE provides calibrated uncertainties that can increase the robustness of applications relying on kinetic parameters.

## Supplementary Material

btae652_Supplementary_Data
